# The effect of zinc-biofortified rice on zinc status of Bangladeshi preschool children: a randomized, double-masked, household-based, controlled trial

**DOI:** 10.1093/ajcn/nqab379

**Published:** 2021-11-18

**Authors:** Roelinda Jongstra, Md Mokbul Hossain, Valeria Galetti, Andrew G Hall, Roberta R Holt, Colin I Cercamondi, Sabina F Rashid, Michael B Zimmermann, Malay K Mridha, Rita Wegmueller

**Affiliations:** Laboratory for Human Nutrition, Institute of Food, Nutrition and Health, ETH Zürich, Zurich, Switzerland; James P Grant School of Public Health, BRAC University, Dhaka, Bangladesh; Laboratory for Human Nutrition, Institute of Food, Nutrition and Health, ETH Zürich, Zurich, Switzerland; Department of Nutrition, University of California, Davis, CA, USA; Department of Nutrition, University of California, Davis, CA, USA; Laboratory for Human Nutrition, Institute of Food, Nutrition and Health, ETH Zürich, Zurich, Switzerland; James P Grant School of Public Health, BRAC University, Dhaka, Bangladesh; Laboratory for Human Nutrition, Institute of Food, Nutrition and Health, ETH Zürich, Zurich, Switzerland; James P Grant School of Public Health, BRAC University, Dhaka, Bangladesh; Laboratory for Human Nutrition, Institute of Food, Nutrition and Health, ETH Zürich, Zurich, Switzerland; GroundWork, Fläsch, Switzerland

**Keywords:** Bangladesh, biofortification, calprotectin, fatty acid desaturases, intestinal fatty acid binding protein, plasma zinc concentration, preschool-age children, rice, zinc, zinc deficiency

## Abstract

**Background:**

Zinc biofortification of rice could sustainably improve zinc status in countries where zinc deficiency is common and rice is a staple, but its efficacy has not been tested. Fatty acid desaturases (FADS) are putative new zinc status biomarkers.

**Objectives:**

Our objective was to test the efficacy of zinc-biofortified rice (BFR) in preschool-aged children with zinc deficiency. Our hypothesis was that consumption of BFR would increase plasma zinc concentration (PZC).

**Methods:**

We conducted a 9-mo, double-masked intervention trial in 12–36-mo-old rural Bangladeshi children, most of whom were zinc-deficient (PZC <70 µg/dL) and stunted (*n* = 520). The children were randomly assigned to receive either control rice (CR) or BFR provided in cooked portions to their households daily, with compliance monitoring. The primary outcome was PZC. Secondary outcomes were zinc deficiency, linear growth, infection-related morbidity, FADS activity indices, intestinal fatty acid binding protein (I-FABP) and fecal calprotectin. We applied sparse serial sampling for midpoint measures and analyzed data by intention-to-treat using mixed-effects models.

**Results:**

At baseline, median (IQR) PZC was 60.4 (56.3–64.3) µg/dL, 78.1% of children were zinc deficient, and 59.7% were stunted. Mean ± SD daily zinc intakes from the CR and BFR during the trial were 1.20 ± 0.34 and 2.22 ± 0.47 mg/d, respectively (*P* < 0.001). There were no significant time-by-treatment effects on PZC, zinc deficiency prevalence, FADS activity, I-FABP, or fecal calprotectin (all *P* > 0.05). There was a time–treatment interaction for height-for-age *z*-scores (*P* < 0.001) favoring the BFR group. The morbidity longitudinal prevalence ratio was 1.08 (95% CI: 1.05, 1.12) comparing the BFR and CR groups, due to more upper respiratory tract illness in the BFR group.

**Conclusions:**

Consumption of BFR for 9 mo providing ∼1 mg of additional zinc daily to Bangladeshi children did not significantly affect PZC, prevalence of zinc deficiency, or FADS activity.

The trial was registered at clinicaltrials.gov as NCT03079583.

## Introduction

Zinc deficiency is a major public health concern in low- and middle-income countries (1) and is associated with higher child morbidity and mortality (2). In Bangladesh, where rice is the main staple food, the risk of inadequate zinc intake is high, due to the low zinc content of white polished rice (3–5). The prevalence of zinc deficiency and stunting in preschool-age children in Bangladesh is 45% and 28%, respectively (6, 7). Preschool-aged children are at high risk of zinc deficiency because their zinc requirement is increased by growth (8).

Zinc biofortification could be a sustainable approach to combat zinc deficiency (9). The bioavailability of zinc-biofortified rice (BFR) is similar to rice fortified with zinc before consumption (10–12). The Bangladesh Rice Research Institute (BRRI) has developed BFR varieties with 28 ppm zinc in polished rice (10, 13) However, efficacy data for these high-zinc rice varieties are lacking.

Assessing the impact of zinc interventions is challenging because of the absence of sensitive and specific biomarkers for zinc status. Measurement of plasma zinc concentration (PZC) is recommended but it might not reflect cellular zinc status and is confounded by many factors (14–16). PZC and zinc intake are often poorly correlated, because PZC is homeostatically controlled over a wide range of zinc intakes (14, 17, 18). A metabolic pathway that could be sensitive to zinc intake is essential fatty acid desaturation (19–21). Both fatty acid desaturase 1 (FADS1) and fatty acid desaturase 2 (FADS2), are zinc-dependent enzymes (1, 22). FADS2 converts linoleic acid (LA; 18:2n–6) to γ-linolenic acid (GLA; C18:3n–6), and FADS1 converts dihomo-γ-linolenic acid (DGLA; C20:3n–6) to arachidonic acid (ARA; C20:4n–6). FADS activities are reflected by their respective conversion ratio (LA:GLA for FADS2, and DGLA:ARA for FADS1) and can respond to changes in dietary zinc in the absence of detectable changes in PZC (23, 24). Plasma intestinal fatty acid binding protein (I-FABP) is a biomarker of intestinal integrity, and fecal calprotectin is a biomarker of intestinal inflammation (25, 26). Intestinal inflammation has been linked to zinc deficiency (27).

The objective of this study was to test the efficacy of BFR to improve zinc status in Bangladeshi preschool-aged children with zinc deficiency and stunting. The primary outcome was PZC. Secondary outcomes included zinc deficiency, FADS, plasma I-FABP, and fecal calprotectin, as well as growth and infection-related morbidity.

## Methods

### Study site

We conducted the study in rural Bangladesh in Badarganj, a subdistrict of Rangpur District that is ∼400 km northwest of the capital, Dhaka. We enrolled participants from 3 unions of Badarganj (Ramnathpur, Bishnupur, and Madhupur).

### Study design and participants

**FIGURE 1:**
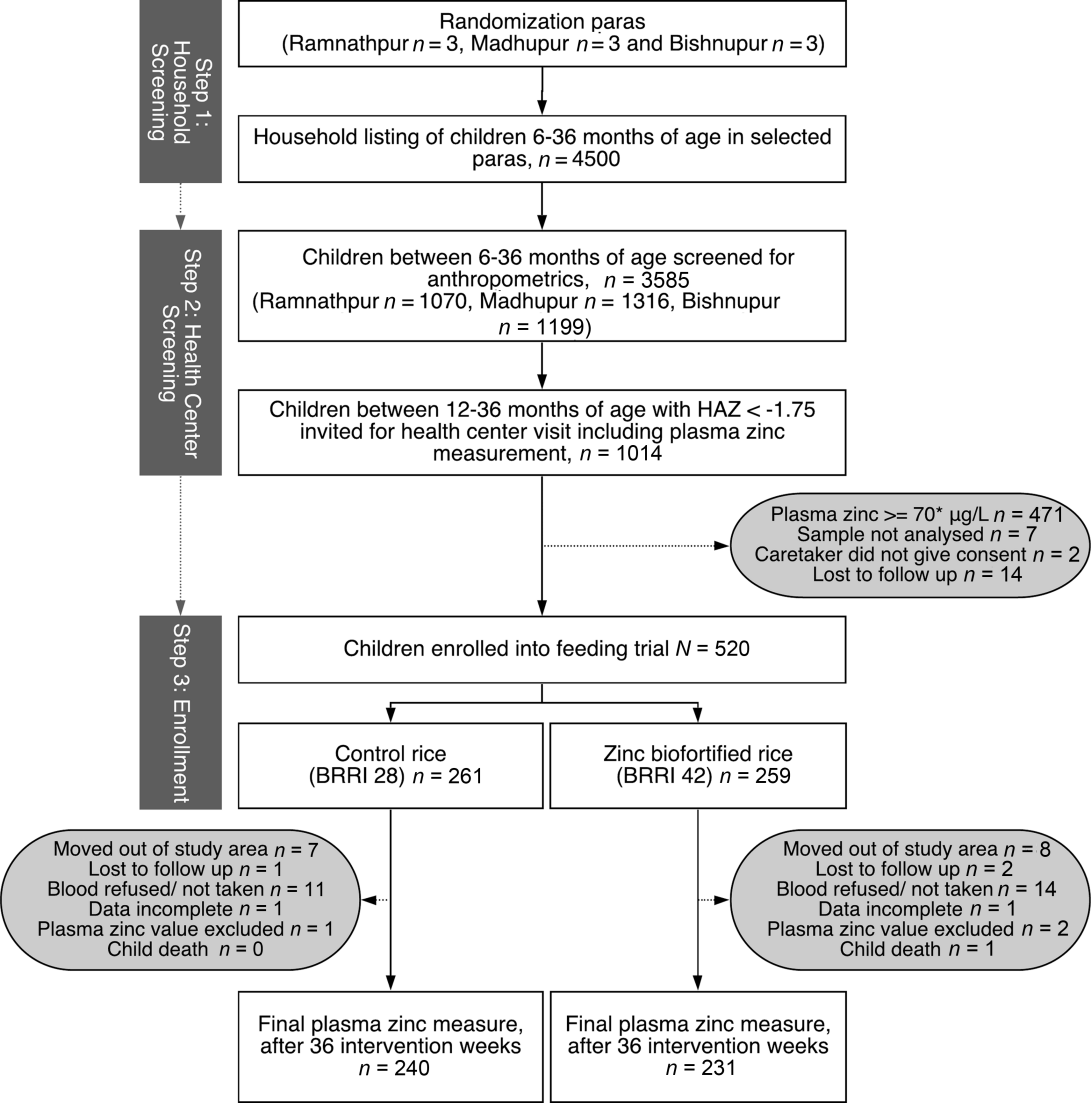
Study diagram. BRRI, Bangladesh Rice Research Institute; HAZ, height-for-age *z*-score.

The study was a 9-mo, double-masked, randomized controlled trial. We began recruiting on April 16, 2018 and completed enrollment on July 9, 2018, and final end-point measurements were completed on April 20, 2019. **Figure 1** shows the study overview; the complete enrollment procedure can be found in the **Supplemental Methods**. In short, the enrollment had 2 screening phases. In the first phase we screened for children's eligibility based on age and children's anthropometrics (height, weight) during household visits (*n* = 3585). For the second phase screening we randomly selected 1014 of these children based on age (12–36 mo) and height-for-age *z*-score (HAZ) < –1.75 (HAZ criterion for stunting was adjusted to meet our sample size calculation; see Supplemental Methods). The second phase took place after sufficient rice harvest was ensured (April 2018) and children were invited to a health center visit in their respective union, during which we collected a venipuncture blood sample to determined hemoglobin (Hb) and PZC.

We enrolled children who met the following inclusion criteria: *1*) 12–36 mo of age (at baseline assessment); *2*) PZC <65 µg/dL, but based on a validation prestudy between the International Centre for Diarrheal Disease Research, Bangladesh (icddr, b) in Dhaka and the Swiss Federal Institute of Technology (ETH), Zurich (data not shown); the PZC cutoff for screening at the icddr, b site was set to <70 µg/dL; *3*) Hb ≥7 g/dL; *4*) no illness or other health conditions that in the opinion of the study team would render the participant unable to comply with the protocol; *5*) no chronic use of medications that affect zinc metabolism; *6*) not taking part in other studies requiring venipuncture; *7*) planning long-term residence at the study site; and *8*) no regular intake (>2 d) of iron-containing mineral and vitamin supplements or fortified foods within the previous 2 mo.

### Ethics

Caregivers of the participating children gave written informed consent twice (first-phase and second-phase screening) in the local language or, in case of illiteracy, oral informed consent after the consent form was read aloud to the caregiver by an independent witness. The study protocol was approved by the ETH Ethics Commission (EK 2017-N-16) and by the Institutional Review Board of Brac University (2017-004). National approval was obtained from the Bangladesh Medical Research Council (BMRC/NREC/2016–2019/294). The study was registered at clinicaltrials.gov with identifier number NCT03079583.

### Randomization and masking

Children meeting all inclusion criteria (*n* = 520) were individually randomly assigned to receive either control rice (CR; *n* = 261) or BFR (*n* = 259) according to a pregenerated sequence inclusion list. They were assigned to 2 randomized sparse serial sampling timepoints (the first from week 3 to 17 and the second from week 18 to 32), generated uniformly between groups with each timepoint set ≥3 wk apart. The end-point sampling date for all children was scheduled in week 36 after 252 feeding days. At each sampling timepoint, primary and secondary outcomes were analyzed except for FADS, I-FABP, calprotectin, and pH, which were only analyzed in baseline and end-point samples. The group assignment was double-masked throughout the study and data analyses used color codes for group identification. An independent person not involved in the study held the study codes. Each child started feeding 21 d after their screening (baseline) visit, to obtain the PZC results from icddr, b.

### Field procedures

In preparation for the health center visits at baseline, the 2 midpoints, and end point, we asked the caregivers to have the children fasted (with the exception of breastfeeding) 12 h before the health center visit and scheduled nearly all visits in the morning (mean time: 11:46 ± 1:06). We followed standardized protocols for blood collection for PZC analysis from the International Zinc Nutrition Consultative Group (28).

A study nurse briefly assessed the child's health to check for eligibility for venipuncture, and the time of last meal consumption was recorded if the child was not fasted. We collected 3 mL whole blood by venipuncture into 2 × 1.5-mL trace element–free lithium heparin tubes (Sarstedt). Hb was directly assessed with a portable HemoCue 201+ photometer (HemoCue AB) using the blood drops from the cannula. All blood tubes were stored on ice and centrifuged within 1 h (3000 × *g*, 10 min, 25°C) on site, after which plasma was aliquoted into 0.1-mL and 0.5-mL acid-washed Eppendorf tubes. We recorded time of blood withdrawal, plasma separation, hemolysis, and turbidity of the aliquots. Aliquoted plasma was stored on ice until transfer to a –20°C freezer within 6 h of separation. After the venipuncture, we provided the child with a small meal (bread, mango juice). Trained field workers measured in duplicate the length (≤2 y of age) or height (>2 y of age) to the nearest 0.1 cm using a length board, and weight to the nearest 0.1 kg using a calibrated scale (Seca 847 digital floor scale) while the children were wearing light clothing and no shoes (29).

The community health workers gave a stool container, spatula, and anaerobic sachet (Microbiology Anaerocult A mini; Merck) to the caregiver 1 wk prior to the scheduled health center visit. Caregivers were trained in collecting a child's stool sample on the morning of the health center visit and requested to bring it with them. The samples were aliquoted in triplicate into 1.5-mL Eppendorf tubes at the end of each day and stored at –20°C. Success rate of collection was 92%.

Community health workers performed morbidity monitoring during weekly household interviews with the caregivers. The interviews were based on standardized symptom questionnaires including fever, cough, respiratory tract infections, vomiting, diarrhea, and potential other symptoms. The onset, severity, and resolution of the symptoms as well as the action taken (e.g., doctor's visit, medication) was reported in the questionnaire. The community health workers administered anthelminthic treatment [albendazole, according to the WHO guidelines (30)] to the participating children before the intervention started and again 6 mo later.

### Rice harvest and analyses

The CR (strain: BRRI28) and BFR (strain: BRRI42) were grown by farmers contracted to the BRRI. To ensure sufficient zinc concentration of the BFR, zinc foliar spraying was applied twice each harvest cycle with zinc sulfate monohydrate (ZnSO_4_H_2_O) at a concentration of 400 g zinc/ha.

After each harvest cycle (*n* = 3), the BRRI delivered CR and BFR in 50-kg color-coded bags to a central storage facility at the field site. The rice was nonparboiled and milled at 7.5%. From the storage facility, color-coded containers of 10 kg rice (head rice to broken rice ratio of 9:1) were delivered to the study kitchens on a weekly basis. For every newly opened rice bag from the rice storage facility (CR *n* = 90, BFR *n* = 93), ∼50-g samples were collected and sent to HarvestPlus in Bogra, Bangladesh, where triplicate X-ray fluorescence measurements of zinc concentration were done to ensure correctness of the color coding by a designated person. Selected rice samples from each batch were shipped to the Human Nutrition Laboratory of ETH Zurich and analyzed for their phytic acid (PA) content by the modified Makower method (31), as previously described in our laboratory (32).

### Rice preparation

Each day, we delivered to the household of each participating child a cooked portion of CR or BFR that had been prepared by trained fieldworkers in the nearest of 9 study kitchens. The rice was cooked in filtered water with a 1:3 rice-to-water ratio. The CR and BFR were cooked separately with different color-coded kitchen utensils. After the rice cooled down, the rice was packed and weighed (with 1-g precision) in unique color/ID-coded food boxes. Standard weight of the rice portions was 240 g but could vary up to 340 g depending on the needs of the child (as requested by the caregiver). The standard weight of 240 g was established based on the mean rice intake assessed by 2-d weighed food records in 12–36-mo-old children (*n* = 72) in a prestudy done in the study area (data not shown). We instructed the caregivers to give the rice ad libitum over the day and they were allowed to add their own side dishes to the rice as well as feed additional foods (e.g., snacks) to the participating children. Caregivers were further instructed to save rice that was not consumed and the study team took back the previous day's rice box containing any leftovers to monitor daily rice consumption.

### Laboratory analyses

During the screening, 1 plasma aliquot (0.5-mL) per child was air-shipped on ice from the field site to icddr, b in Dhaka, for baseline PZC measurement on a weekly basis. PZC was determined in duplicate by using flame atomic absorption spectrometry, and C-reactive protein (CRP) was measured by using an immunoassay (Tina-quant CRP Gen.3; Roche). At the completion of the study, all samples were shipped frozen on dry ice to the Human Nutrition Laboratory of ETH Zurich. Analyses of plasma ferritin (PF), CRP, and α-1-acid glycoprotein (AGP) were done using an immunoassay (33). PZC was analyzed by using inductively coupled plasma MS (iCAP; Thermo-Fisher Scientific), for all timepoints including baseline. After thawing, 200 µL plasma was pipetted in duplicate into 10-mL polypropylene tubes and 4.8 mL zinc plasma diluent composed of 0.5% v/v nitric acid, 1% propanol, 0.05% Triton X, and ultrapure water (>18.2 MΩ) was added. We included 1 duplicate control sample with Seronorm Trace Element Serum L-2 (Sero AS) in every sample run. All used material was trace element–free or acid-washed. We repeated the analyses if the duplicate's CV was >15%. We calculated the prevalence of zinc deficiency as PZC <65 µg/dL (34). Subclinical inflammation was defined as CRP >5 mg/L and/or AGP >1 g/L as a possible confounder for PZC (35). Iron deficiency was defined as PF <12 µg/L when adjusted for inflammation (36), and anemia as Hb <11 g/dL (37, 38). For the secondary analyses at screening and end point we measured FADS in a random subsample of 150 children (*n* = 75 per study arm) with matched nonhemolyzed plasma with sufficient volume. Determination of a panel of total plasma fatty acids (FAs) was done by the OmegaQuant Laboratory using LC tandem MS, following extraction and analytical methods previously reported (39). Another subsample of 120 children (∼60 per study arm) was used to measure plasma I-FABP, fecal calprotectin, and fecal pH. This subsample was selected based on sufficient sample material (stool sample and nonhemolyzed plasma) and was not further randomized as sufficient plasma was a limiting factor. Plasma I-FABP and fecal calprotectin was measured by ELISAs (respectively HK406, Hycult Biotech; and Calprest NG, Eurospital) and fecal pH as previously reported by our laboratory (40). For fecal calprotectin, values >50 mg/kg were considered elevated (Calprest NG; Eurospital). For I-FABP and calprotectin, samples with CV >15% were excluded from analyses (*n* = 11 and 9, respectively). I-FABP measurements below the limit of detection (<47.0 pg/nL) were randomly allocated a value between 0 and 47.0 (*n* = 47). Calprotectin measurements below the limit of detection (<27 mg/kg) were randomly allocated a value between 0 and 27 (*n* = 43).

### Statistical analysis

The sample size calculation was based on a previous zinc rice fortification trial reporting a 5% difference in PZC (3.3 µg/dL) when comparing the intervention group with the control group (41). Using the relatively high SD of this previous trial at end point (8.5 µg/dL), the power calculation indicated that 141 children would be needed in each group to detect a difference of 3.3 µg/dL in mean PZC with a significance level of 0.05 (2-tailed) and 90% power. Taking into account that this previous trial was a fortification trial with higher between-group difference in additional daily zinc intake (8 mg compared with ∼1 mg anticipated in our intervention, based on 12 ppm difference between control and biofortified intervention rice) we increased the sample size by 50%, resulting in 211 children per group. Anticipating a dropout rate of 10%, we aimed to include 235 children per study group. Finally, to be able to equally divide the study population over the selected unions, we decided to include 260 children per study arm (total *n* = 520).

HAZ, weight-for-age *z*-scores (WAZ), and weight-for-height *z*-scores (WHZ) were calculated using the WHO Stata macro (42). For morbidity data, longitudinal prevalence ratios (LPRs) and 95% CIs were calculated from weekly 1-d recalls. We performed statistical analyses with Stata (version 16.0; StataCorp LLC). During a validation prestudy (*n* = 60), we found discrepancies between the PZC measured at icddr, b and ETH (21% overestimation at the icddr, b site, data not shown) and we therefore repeated baseline PZC at ETH. However, for 69 (13%) children we had insufficient baseline plasma to repeat PZC analysis. For these children we used a regression model to impute the PZC found at icddr, b; the model was based on PZC measured at both icddr, b and ETH Zurich and included 819 matched data points at baseline. The model for multiple PZC imputation was:

(1) \begin{eqnarray*} PZC\,\,imputed\left( {\frac{{\mu g}}{{dL}}} \right) = 8.816 + 0.80 \times PZC\,\,ICDDR,B\left( {\frac{{\mu g}}{{dL}}} \right) \end{eqnarray*}

and the resulting values were used for the intention-to-treat (ITT) analysis. We did not perform any imputation for secondary outcomes. Median PZC values adjusted for clinical infection using BRINDA (Biomarkers Reflecting Inflammation and Nutritional Determinants of Anemia) did not differ from unadjusted values and therefore the latter values were used in all analyses (43). For the FADS analysis, all FAs were expressed in micromoles per liter, and percentage FAs calculated from the sum of all measured FAs in micromoles. When data were not normally distributed according to the Shapiro–Wilk test, values were transformed using natural logarithms before statistical analysis. Values in the text and in the tables are presented as mean ± SD for normally distributed data, median (IQR) for nonnormal data, and percentage (95% CI) for prevalences. Baseline characterisitcs were not compared between groups (**Table 1**). Rice zinc concentration data were analyzed using 1-factor ANOVA for continuous data and Pearson χ ^2^ tests for percentage data (**Table 2**). Anthropometrics, zinc, iron, inflammation markers, FA concentrations, and gut inflammation data during the intervention were analyzed using mixed-effects model (MEM) for continuous data and logistic regression MEM for binary outcomes. Fixed effects on the dependent variables were time (defined as study day from intervention start to account for subject-specific sampling intervals), treatment (defined as the randomization group), and their interaction (time-by-treatment effect). Subject was the random component (**Tables 3** and **4**). Morbidity data were analyzed using LPR (**Table 5**). In addition, we performed per protocol (PP) analysis using the above described MEM model (**Supplemental Table 1**). For the PP analysis we included all children who were zinc deficient at baseline (measured at ETH Zurich, no imputation), who had a valid plasma zinc end-point measure, and with high compliance to the intervention (≥202 d of rice intake data). For all models, heteroscedasticity was carefully checked by visual inspection of the residuals on the Tukey–Anscombe plots. Significance of all tests was set at *P* < 0.05.

## Results

### Baseline characteristics

Table 1 shows the baseline characteristics of the 2 groups. From all included children (PCZ <70 µg/dL, as measured at icddr, b and described above), only 78% had PZC <65 µg/dL based on the measurements performed at ETH. From the Household Food Insecurity Access Scale scores, both groups reported ∼60% food security.

**TABLE 1:** Baseline characteristics of enrolled preschool children aged 1–3 y (*n* = 520) in total and by study group^1^

	*n*	Total	*n*	CR	*n*	BFR
Child sex, age, and anthropometrics						
Sex, % male/*n*	520	56.7/295	261	57.1/149	259	56.4/146
Age, mo	518	25.0 (19.0–30.0)	260	25.0 (19.0–30.5)	258	25.0 (19.0–29.0)
Height, cm	518	80.8 (75.8–84.2)	260	80.9 (75.9–84.3)	258	80.5 (75.7–84.0)
Weight, kg	518	9.5 (8.5–10.5)	260	9.5 (8.5–10.5)	258	9.4 (8.5–10.5)
WHZ	518	−1.2 ± 0.8	260	−1.2 ± 0.9	258	−1.1 ± 0.8
Wasting, WHZ ←2 (%/*n*)		16.8/87		19.2/50		14.3/37
HAZ	516	−2.2 ± 0.8	259	−2.2 ± 0.7	257	−2.2 ± 0.8
Stunting, HAZ ←2 (%/*n*)		59.7/308		58.3/151		61.1/157
WAZ	518	−2.0 ± 0.8	260	−2.0 ± 0.8	258	−2.0 ± 0.8
Underweight, WAZ ←2 (%/*n*)		49.8/258		52.3/136		47.3/122
Breastfeeding (%/*n*)	520	75.6/384	261	77.7/198	259	73.5/186
Zinc, iron, and inflammation status markers						
PZC,^2^ µg/dL	520	60.4 (56.3–64.3)	261	60.8 (56.5–64.8)	259	59.9 (56.0–63.8)
Zinc deficiency <65 µg/dL (%/*n*)		78.1/406		75.9/198		80.3/208
PF adj,^3^ µg/L	483	29.6 (15.6–45.7)	242	29.3 (14.6–45.6)	241	30.8 (16.5–45.7)
Iron deficiency, PF adj <12 µg/L (%/*n*)		17.4/84		19.4/47		15.4/37
Hb, g/dL	517	11.0 (10.2–11.8)	259	10.8 (10.2–11.6)	258	11.2 (10.4–11.9)
Anemia, Hb <11 g/dL (%/*n*)		47.8/247		54.4/141		41.1/106
CRP, mg/L	483	0.4 (0.1–2.5)	242	0.4 (0.1–2.5)	241	0.4 (0.1–2.1)
Inflammation, CRP >5 mg/L (%/*n*)		15.9/77		14.5/35		17.4/42
AGP, g/L	483	0.7 (0.5–1.0)	242	0.7 (0.5–0.9)	241	0.7 (0.5–1.0)
Inflammation, AGP >1 g/L (%/*n*)		21.7/105		19.4/47		24.1/58
Household socioeconomic characteristics						
Mother's age, y	520	24.0 (21.0–28.0)	261	24.0 (20.0–27.0)	259	24.0 (21.0–28.0)
Religion (%/*n*)						
Islam		88.7/461		86.6/226		90.7/235
Hinduism		10.6/55		13.0/34		8.1/21
Christianity		0.8/4		0.4/1		1.2/3
Mother's education level (%/*n*)						
Never attended school		10.2/53		11.1/29		9.3/24
Primary		68.1/354		65.9/172		70.3/182
Secondary		19.2/100		20.7/54		17.8/46
Tertiary		2.5/13		2.3/6		2.7/7
Household food insecurity score (%/*n*)						
Food secure		63.1/328		64.4/168		61.8/160
Mildly food insecure		11.0/57		9.2/24		12.7/33
Moderately food insecure		18.1/94		18.8/49		17.4/45
Severely food insecure		7.8/41		7.7/20		8.1/21

1 Normal data are mean ± SD, nonnormal data are median (IQR), or prevalence data are %/*n*. AGP, α-1-acid glycoprotein; BFR, zinc-biofortified rice; CR, control rice; CRP, C-reactive protein; HAZ, height-for-age *z*-score; Hb, hemoglobin; PF, plasma ferritin; PZC, plasma zinc concentration; WAZ, weight-for-age *z*-score; WHZ, weight-for-height *z*-score.

2 Based on measurements performed at the Swiss Federal Institute of Technology (ETH), Zurich.

3 Plasma ferritin adjusted (PF adj) for inflammation (36).

### Rice intake and rice zinc concentration

Average rice consumption and the corresponding zinc intakes are shown in Table 2. One child moved out of the study area before the start of the intervention, and 3 children had no available rice intake data. Another 29 children (CR, *n* = 16; BFR, *n* = 13) received the wrong rice during the whole intervention period. All were treated per ITT and maintained in their randomized group, and were only allocated to the correct treatment group for PP analyses. Total feeding days and total amount of rice consumed did not differ between the groups. Moreover, daily mean rice consumption was similar in the CR and BFR groups (232.7 ± 49.8 g/d; 239.1 ± 43.4 g/d, respectively). There was a significant between-group difference in mean daily zinc intake: 1.20 ± 0.34 mg/d in the CR group and 2.22 ± 0.47 mg/d in the BFR group (*P* < 0.001) (Table 2).

**TABLE 2:** Rice zinc and phytic acid content and zinc intake by study group^1^

	Total (*n* = 520 included at baseline)	Control rice (*n* = 261 included at baseline)	Zinc-biofortified rice (*n* = 259 included at baseline)	*P* value
Zinc,^2^ µg/g raw rice	—	14.7 ± 0.8	28.5 ± 3.5	<0.001
Phytic acid,^3^ mg/g raw rice	—	3.02 ± 0.12	3.71 ± 0.06	0.333
Phytic acid:zinc molar ratio	—	20.36	12.89	
Total feeding days, *n* (± SE)	118,712 ± 883	59,339 ± 685	59,373 ± 558	0.579
Total rice consumption, kg cooked rice (± SE)	28,349 ± 317	14,032 ± 232	14,316 ± 215	0.214
Average feeding days, no. of feeding days/child	228.3 ± 38.7	227.4 ± 42.4	229.2 ± 34.7	0.579
Average rice intake, g cooked rice/d/child	235.9 ± 46.8	232.7 ± 49.8	239.1 ± 43.4	0.119
Average zinc intake from study rice, mg/total feeding period/child	394.4 ± 164.6	277.3 ± 90.0	512.4 ± 136.0	<0.001
Average zinc intake from study rice, mg/d/child	1.71 ± 0.65	1.20 ± 0.34	2.22 ± 0.47	<0.001
Compliance^4^ (%/*n*)	81.9/426	83.5/218	80.3/208	0.341

1 Data are mean ± SD unless stated otherwise. Between-group difference tested using 1-factor ANOVA for continuous data and Pearson χ^2^ test for prevalence data.

2 Based on average zinc content (µg/g) from X-ray fluorescence analyses performed at Harvest Plus Bogra; control rice bags *n* = 96, biofortified rice bags *n* = 92.

3 Based on average phytic acid (mg/g) of rice varieties from each harvest batch (*n* = 3) sent to the Swiss Federal Institute of Technology (ETH), Zurich.

4 Compliance based on percentage of children consuming 80% of their total calculated study rice portion for total intervention time (intervention time 252 d; rice portion per day: age ≤24 mo 150 g cooked/50 g raw rice, >24 mo 240 g cooked/80 g raw rice).

### Plasma zinc concentration and prevalence of zinc deficiency

**FIGURE 2:**
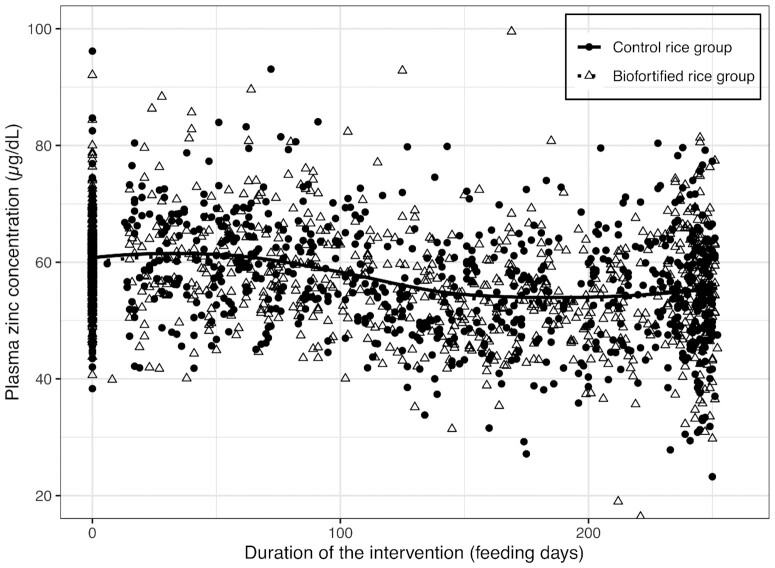
Kinetic curves of plasma zinc concentration during the 36-wk intervention in Bangladeshi children, by group (control rice group *n* = 240, biofortified rice group *n* = 231). Lines show the fitted values using a local polynomial regression fit (function LOESS). Six data points were excluded that were outside the plasma zinc concentration range of 20–100 µg/dL.

PZC declined in the CR and BFR groups over the study, and the prevalence of zinc deficiency increased by 8.7% and 3.7% in the CR and BFR groups, respectively (**Figure 2**, Table 3). The crude MEM showed no significant interaction predictor between treatment and time (*P* = 0.282) but an association with time (*P* < 0.001) (Table 3). For the PP analyses, 65% of enrolled children were included (CR, *n* = 165; BFR, *n* = 172) and findings were similar (Supplemental Table 1). The baseline to end-point change in PZC was negatively predicted by baseline PZC (CR, –0.77 µg/dL; BFR, –0.97 µg/dL change per µg/dL higher baseline PZC) (**Supplemental Figure 1**).

**TABLE 3:** Anthropometrics, zinc, iron, and inflammation markers for the total population (*n* = 520), and intestinal inflammation markers for the subsample (*n* = 120) by study group at baseline to end point. Total weeks of intervention *n* = 36^1^

	Control rice		Zinc-biofortified rice		MEM model		
	*n*		*n*		*P* ^2^	*P* ^3^	*P* ^4^
Zinc, iron, and inflammation status markers							
PZC,^5^ µg/dL							
Baseline	261	60.8 (56.5–64.8)	259	59.9 (56.0–63.8)			
End point	240	55.1 (48.3–62.3)	231	55.6 (49.1–63.0)	<0.001	0.276	0.282
Zinc deficiency <65 µg/dL (%/*n* )							
Baseline		75.9/198		80.3/208			
End point		84.6/203		84.0/194	<0.001	0.043	0.139
PF adj,^6^ µg/L							
Baseline	242	29.3 (14.6–45.6)	241	30.8 (16.5–45.7)			
End point	238	30.4 (20.0–45.8)	233	33.2 (23.3–46.5)	0.109	0.834	0.205
Iron deficiency, PF adj <12 µg/L (%/*n*)							
Baseline		19.4/47		15.4/37			
End point		10.5/25		7.7/18	0.002	0.49	0.899
Hb, g/dL							
Baseline	259	10.8 (10.2–11.6)	258	11.2 (10.4–11.9)			
End point	237	11.3 (10.7–11.8)	237	11.3 (10.7–12.0)	<0.001	0.037	0.14
Anemia, Hb <11 g/dL (%/*n*)							
Baseline		54.4/141		41.1/106			
End point		34.2/81		32.9/78	<0.001	0.02	0.024
CRP, mg/L							
Baseline	242	0.4 (0.1–2.5)	241	0.4 (0.1–2.1)			
End point	238	0.1 (0.0–0.9)	233	0.1 (0.0–0.9)	<0.001	0.951	0.925
Inflammation, CRP >5 mg/L (%/*n*)							
Baseline		14.5/35		17.4/42			
End point		6.7/16		9.8/23	0.009	0.458	0.965
AGP, g/L							
Baseline	242	0.7 (0.5–0.9)	241	0.7 (0.5–1.0)			
End point	238	0.6 (0.4–0.8)	233	0.6 (0.4–0.8)	0.003	0.893	0.96
Inflammation, AGP >1 g/L (%/*n*)							
Baseline		19.4/47		24.1/58			
End point		13.0/31		14.6/34	0.062	0.56	0.676
Anthropometrics							
WHZ							
Baseline	260	−1.2 ± 0.9	258	−1.1 ± 0.8			
End point	251	−0.9 ± 0.8	246	−0.5 ± 6.4	0.079	0.878	0.161
Wasting, WHZ ←2 (%/*n*)							
Baseline		19.2/50		14.3/37			
End point		10.8/27		8.6/21	<0.001	0.102	0.601
HAZ							
Baseline	259	−2.2 ± 0.7	257	−2.2 ± 0.8			
End point	251	−2.1 ± 0.8	245	−2.0 ± 0.7	<0.001	0.351	0.001
Stunting, HAZ ←2 (%/*n*)							
Baseline		58.3/151		61.1/157			
End point		53.0/133		50.2/123	0.069	0.883	0.066
WAZ							
Baseline	260	−2.0 ± 0.8	258	−2.0 ± 0.8			
End point	251	−1.8 ± 0.8	246	−1.7 ± 0.8	<0.001	0.138	0.079
Underweight, WAZ ←2 (%/*n*)							
Baseline		52.3/136		47.3/122			
End point		40.2/101		36.2/89	<0.001	0.256	0.466
Breastfeeding (%/*n*)							
Baseline		77.7/198		73.5/186	<0.001	0.427	0.252
End point		47.2/119		47.1/117			
Intestinal markers							
I-FABP, pg/mL							
Baseline	55	81.1 (47.9–138.4)	51	63.5 (59.7–102.6)			
End point	44	80.0 (43.1–127.3)	43	107.9 (72.4–191.5)	0.775	0.626	0.183
Fecal calprotectin, mg/kg							
Baseline	58	217.1 (98.2–603.5)	54	179.8 (44.3–304.6)			
End point	58	97.2 (42.8–264.0)	55	128.9 (23.5–546.3)	0.021	0.066	0.078
Fecal pH							
Baseline	61	5.3 (5.1–5.8)	59	5.2 (5.0–5.7)			
End point	61	5.4 (5.2–6.0)	59	5.5 (5.0–5.8)	0.240	0.519	0.963

1 Normal data are mean ± SD, nonnormal data are median (IQR), or prevalence data are %/*n*. Data were analyzed using a mixed-effects model (MEM) for continuous data as dependent variable, or logistic regression MEM for binary outcomes as dependent variable. All models used time (study day from intervention start), treatment (CR compared with BFR), and their interaction as fixed effects. Subject was used as the random effect. The models contained all sampling time points (baseline to end point and 2 mid sparse random sampling points) except subsample analyses containing only baseline and end-point data. Significance was set at *P* < 0.05. AGP, α-1-acid glycoprotein; BFR, zinc-biofortified rice; CR, control rice; CRP, C-reactive protein; HAZ, height-for-age *z*-score; Hb, hemoglobin; I-FABP, intestinal fatty acid binding protein; MEM, mixed-effects model; PF, plasma ferritin; PZC, plasma zinc concentration; WAZ, weight-for-age *z*-score; WHZ, weight-for-height *z*-score.

2 Testing main effect of time.

3 Testing main effect of treatment.

4 Testing effect of time-by-treatment interaction.

5 Based on measurements performed at the Swiss Federal Institute of Technology (ETH), Zurich.

6 Plasma ferritin adjusted for inflammation (PF adj) (36).

### Anthropometrics, iron, and inflammation status

The effect of the intervention on anthropometrics, iron, or inflammation biomarkers is presented in Table 3. Prevalence of wasting, underweight, and stunting declined over the study in both groups, and time was a significant predictor of the prevalence of wasting and underweight as well as HAZ and WAZ (*P* < 0.001 for all). There was no significant treatment effects for any of the anthropometric indicators but there was a time-by-treatment interaction for HAZ (*P* < 0.001). Prevalence of iron deficiency decreased over the study by 8.9% in the CR group and 7.7% in the BFR group; time was a significant predictor (*P* = 0.002). Hb concentration increased and anemia prevalence decreased over the study, with significant time and treatment effects for both, and a time-by-treatment interaction for anemia. Anemia prevalence dropped by 20.2% in the CR group and 8.2% in the BFR group. Inflammation decreased over the study, and time was a significant predictor of CRP concentration (*P* < 0.001), prevalence of elevated CRP (*P* = 0.009), and AGP concentration (*P* = 0.003).

### Plasma I-FABP, fecal calprotectin, and pH

The MEM analyses showed no association of plasma I-FABP, fecal calprotectin, and pH with treatment or time, and there was no significant interaction predictor between treatment and time, with the exception of a time effect for fecal calprotectin, which fell in both groups over the study (*P* = 0.021) (Table 3).

### FADS

Baseline and end-point FA concentrations as percentage of total FAs are shown in Table 4. At baseline, the LA: ɑ-linolenic acid (ALA; C18:3n–3) ratio was significantly higher in the CR group compared with the BFR group (*P* = 0.048), whereas docosapentaenoic acid (DPA; 22:5n–3) was higher in the BFR group compared with the CR group (*P* = 0.026). From the MEM analyses, time was a significant predictor (*P* < 0.01) for all FA concentrations except for the combined activity indices of elongase/δ-6-desaturase/peroxisomal β-oxidation (ELOV/D6D/SPCS; C22:6n–3/22:5n–3). Treatment was a significant predictor (*P* = 0.033) for DPA but there were no significant time-by-treatment effects (Table 4).

**TABLE 4:** Fatty acid concentrations (% total FA) by study group at baseline and end point, based on a subsample (*n* = 150) of the total study population (*n* = 520). Total weeks of intervention *n* = 36^1^

	CR	BFR	MEM model		
	*n* = 75	*n* = 75	*P* ^2^	*P* ^3^	*P* ^4^
SFA and MUFA					
C16:0 (palmitic)					
Baseline	31.2 (6.4)	30.9 (6.3)			
End point	28.3 (11.8)	27.9 (8.4)	<0.001	0.381	0.841
C16:1n–7 (palmitoleic)					
Baseline	1.78 (4.66)	1.81 (3.68)			
End point	1.43 (3.29)	1.45 (2.68)	<0.001	0.81	0.983
C18:0 (stearic)					
Baseline	8.24 (4.70)	8.21 (2.75)			
End point	7.23 (3.88)	7.29 (3.28)	<0.001	0.767	0.271
C18:1n–9 (oleic)					
Baseline	25.6 (13.6)	25.5 (11.9)			
End point	23.6 (18.8)	23.2 (15.5)	<0.001	0.793	0.701
C24:1n–9 (nervonic)					
Baseline	0.44 (0.97)	0.40 (0.04)			
End point	0.24 (0.82)	0.24 (0.75)	<0.001	0.492	0.227
Omega-6 PUFA					
C18:2n–6 (LA)					
Baseline	23.4 (16.0)	23.6 (15.4)			
End point	27.8 (16.2)	28.4 (14.0)	<0.001	0.752	0.745
C18:3n–6 (GLA)					
Baseline	0.14 (0.46)	0.16 (0.37)			
End point	0.28 (0.92)	0.30 (0.98)	<0.001	0.095	0.454
C20:3n–6 (DGLA)					
Baseline	0.80 (0.93)	0.82 (0.85)			
End point	0.90 (1.09)	0.95 (1.06)	0.001	0.717	0.517
C20:4n–6 (ARA)					
Baseline	3.08 (2.84)	3.15 (3.96)			
End point	4.29 (5.63)	4.58 (5.74)	<0.001	0.633	0.332
C22:4n–6 (adrenic)					
Baseline	0.12 (0.20)	0.12 (0.25)			
End point	0.14 (0.25)	0.15 (0.20)	0.009	0.523	0.35
C22:5n–6 (DPA n–6)					
Baseline	0.12 (0.25)	0.12 (0.21)			
End point	0.17 (0.30)	0.17 (0.40)	<0.001	0.862	0.667
∑n–6					
Baseline	28.0 (16.0)	28.3 (17.6)			
End point	34.0 (20.6)	34.9 (17.4)	<0.001	0.585	0.585
Omega-3 PUFA					
C18:3n–3 (ALA)					
Baseline	0.19 (0.44)	0.21 (0.39)			
End point	0.33 (0.93)	0.34 (0.67)	<0.001	0.058	0.236
C20:5n–3 (EPA)					
Baseline	0.10 (0.35)	0.11 (0.26)			
End point	0.14 (0.73)	0.16 (2.13)	<0.001	0.382	0.659
C22:5n–3 (DPA n-3)					
Baseline	0.11 (0.25)	0.13 (0.40)*			
End point	0.18 (0.44)	0.21 (0.54)	<0.001	0.033	0.732
C22:6n–3 (DHA)					
Baseline	0.58 (0.95)	0.60 (1.19)			
End point	0.93 (1.40)	1.02 (2.11)	<0.001	0.605	0.306
∑n–3					
Baseline	1.01 (1.60)	1.09 (1.41)			
End point	1.65 (2.74)	1.78 (4.79)	<0.001	0.116	0.965
∑n–6:∑n–3					
Baseline	2.09 (0.96)	2.04 (0.80)			
End point	20.7 (25.1)	19.6 (24.2)	<0.001	0.118	0.821
LA:ALA					
Baseline	27.7 (29.0)	25.9 (28.1)*			
End point	84.0 (171.2)	83.0 (133.9)	<0.001	0.064	0.166
Activity indices					
SCD1 16:1n–7/16:0					
Baseline	0.06 (0.14)	0.06 (0.12)			
End point	0.05 (0.11)	0.05 (0.09)	0.008	0.705	0.953
SCD1 18:1n–9/18:0					
Baseline	3.10 (2.81)	3.10 (2.09)			
End point	3.26 (4.49)	3.18 (3.21)	0.011	0.97	0.415
D6D 18:3n–6/18:2n–6					
Baseline	0.006 (0.026)	0.007 (0.023)			
End point	0.01 (0.03)	0.01 (0.04)	<0.001	0.128	0.414
D5D 20:4n–6/20:3n–6					
Baseline	3.84 (8.11)	3.87 (6.06)			
End point	4.75 (7.77)	4.84 (8.59)	<0.001	0.943	0.808
ELOV1/6 C18:0/C16:0					
Baseline	0.27 (0.16)	0.27 (0.12)			
End point	0.26 (0.23)	0.26 (0.16)	0.008	0.829	0.383
ELOV2 22:5n–3/20:5n–3					
Baseline	1.09 (3.68)	1.17 (3.40)			
End point	1.32 (3.90)	1.33 (4.00)	0.036	0.405	0.534
ELOV2 22:4n–6/20:4n–6					
Baseline	0.04 (0.11)	0.04 (0.09)			
End point	0.03 (0.06)	0.03 (0.05)	0.001	0.374	0.791
ELOV/D6D/SPCS C22:5n–6/22:4n–6					
Baseline	0.96 (2.01)	0.99 (2.75)			
End point	1.16 (1.85)	1.14 (2.90)	0.005	0.709	0.713
ELOV/D6D/SPCS C22:6n3/22:5n3					
Baseline	5.24 (9.76)	4.63 (10.7)			
End point	4.93 (13.2)	4.99 (11.9)	0.660	0.068	0.251

1 Data presented as the geometric mean (range). *Significant between-group differences assessed by pooled *t* test (*P < 0.05). A mixed-effects model (MEM) used fatty acids as dependent variable (ln transformed), and time (study day from intervention start), treatment (CR compared with BFR), and their interaction as fixed effects. Subject was used as the random effect. Significance was set at *P* < 0.05. ALA, α-linolenic acid; ARA, arachidonic acid; BFR, zinc-biofortified rice; CR, control rice; DGLA, dihomo-ƴ-linolenic; DHA, dosahexaenoic; DPA, docosapentaenoic; D5D, Δ^5^-desaturase; D6D, Δ^6^-desaturase; EPA, eicosapentaenoic; ELOV, elongase; GLA, γ-linolenic acid; LA, linoleic acid; MEM, mixed-effects model; SCD, stearoyl-CoA desaturase; SPCS, peroxisomal β-oxidation; ∑n–3, sum of measured ω-3 fatty acids; ∑n–6, sum of measured ω-6 fatty acids.

2 Testing main effect of time.

3 Testing main effect of treatment.

4 Testing effect of time-by-treatment interaction.

### Morbidity

Morbidity rates by group and by symptom collected over 36 weekly household visits are shown in Table 5. For total morbidity, the LPR was 1.08 (95% CI: 1.05, 1.12) comparing the BFR and the CR groups. Respiratory tract morbidity was also greater in the BFR group compared with the CR group: LPR was 1.09 (95% CI: 1.06, 1.12) for colds and 1.26 (95% CI: 1.08, 1.46) for difficulty in breathing/wheezing.

**TABLE 5:** Longitudinal prevalence of morbidity by group based on data from weekly household visits (*n* = 36) assessing child health status on the previous day^1^

	Days with illness (*n*)	Days of observation (*n*)	% LP	LPR	95% CI
Any of the symptoms present yesterday:					
Fever					
BFR	1330	8950	14.86	1.04	0.97, 1.11
CR	1284	8967	14.32		
Cold					
BFR	4366	8932	48.88	1.09	1.06, 1.12
CR	4015	8950	44.86		
Chest problem^2^					
BFR	371	8949	4.15	1.26	1.08, 1.46
CR	295	8967	3.29		
Vomiting^3^					
BFR	135	8947	1.51	1.02	0.81, 1.30
CR	132	8962	1.47		
Diarrhea^4^					
BFR	53	8926	0.59	0.90	0.62, 1.30
CR	59	8939	0.66		
Total morbidity					
BFR	6255	44,704	13.99	1.08	1.05, 1.12
CR	5785	44,785	12.92		

1 CR, control rice; BFR, zinc-biofortified rice; % LP, longitudinal prevalence of morbidity; LPR, longitudinal prevalence ratio with corresponding 95% CI.

2 Difficulty breathing, wheezing.

3 Vomiting (≥3 times).

4 Diarrhea (≥3 loose stools).

## Discussion

The main findings of this study are that consumption by Bangladeshi preschool children of BFR providing ∼1 mg additional zinc daily for 9 mo, compared with CR: *1*) did not significantly affect PZC or the prevalence of zinc deficiency; *2*) did not significantly affect FADS activity, I-FABP, or fecal calprotectin; and *3*) significantly increased overall risk of infection-related morbidity, particularly of the upper respiratory tract.

In the recent Micronutrient Survey in Bangladesh, the national prevalence of zinc deficiency (PZC <65 µg/dL) in preschool age children was 45% (7), and the median dietary zinc intake was 3.2 mg/d (35). Our own dietary intake data using weighed food records prior to the study as well as 24-h recalls conducted during the first 3 mo of the study suggest median zinc intakes of 2.9–3.0 mg/d (data not shown), which is in line with other reported studies (6, 44). These intakes all approximate the RDA of 3 mg/d (45, 46). Although the impact of phytic acid on zinc absorption might be limited in children, the bioavailability of zinc from rice based on their PA:zinc molar ratio was 23% for the CR group and 31% for the BFR group (45, 47). Altogether, the suggested adequate zinc intake might not cover the potential higher needs in this population because of impaired absorption due to frequent infections (48) and low zinc bioavailability from the diet and thus might explain the lack of correlation between zinc intake and PZC.

Our findings of a lack of an intervention effect on PZC are consistent with several previous studies (49, 50). A systematic review (51) concluded that zinc fortification at low levels (between 4 and 10 mg/d) had no significant effect on PZC, whereas zinc supplementation at similar levels increased PZC and reduced the risk of zinc deficiency (51). In contrast, a recent systematic review concluded that zinc fortification (single or with multiple micronutrients) increases plasma zinc concentrations (52). In a recent pooled analysis of 3 postharvest zinc-fortified rice studies, the nonsignificant mean between-group difference was 0.38 μmol/L (53). Because postharvest zinc fortification shows similar zinc absorption patterns as zinc-biofortified rice (10), the low amount of zinc provided by the BFR in our study (∼1 mg/d) could have contributed to the lack of effect.

PZC is the only zinc biomarker recommended to assess zinc status and is a useful biomarker of severe, but not of mild-to-moderate, zinc deficiency (14, 17, 54). Therefore, we explored the potential of FADS as a new biomarker of zinc intake (23). We did not find a treatment or time-by-treatment interaction effect on FADS1 or FADS2 activities. This suggests the additional zinc (∼1 mg/d) provided through the BFR is insufficient to influence FADS activity. Similar outcomes were reported from other studies, in which FADS activities did not respond to 2.8 mg of additional zinc from zinc-fortified water (20). Another study reported changes in FADS activities with zinc-biofortified wheat intake (6.3 mg additional zinc), although the effect might be attributed to the study design, as suggested by the authors (55).

The decrease in PZC over our study in both groups could have been due to seasonal changes in zinc intake from the habitual diet. Seasonality in Bangladesh is known to affect household food security and dietary diversity; these typically worsen during the monsoon season (56, 57). However, our end-point measures were done in the dry season and lower dietary diversity was not expected; moreover, there was an improvement in iron and anemia status during the study in both groups, suggesting that diets did not worsen during this period. This latter finding could have been at least partly due to deworming of the children. Considering the poor sanitation in the area, the children in our study could have had environmental enteric dysfunction (EED), and EED is known to affect zinc absorption in Bangladeshi preschool children (58, 59). We did not assess EED, but median fecal calprotectin in both groups and at all timepoints was above the assay cutoff suggesting the presence of intestinal inflammation (25, 60, 61). However, plasma I-FABP was not elevated in most children, suggesting enterocyte integrity was largely intact (62). Two studies in preschool children from Bangladesh and Brazil reported higher I-FABP values than in our study (63, 64).

In children, severe zinc deficiency can weaken immune function and increase risk of respiratory infections, malaria, and diarrhea (53). In recent reviews (51, 65), provision of zinc doses <10 mg/d had no clear effect on fever and respiratory tract infections in children aged <5 y (65), and showed mixed results on diarrhea (51, 65). Our intervention had no significant effect on diarrhea, and we do not have any plausible explanation for the 8% higher overall morbidity in the BFR group—it could be a chance finding.

The strengths of our study include: *1*) its double-masked, randomized controlled design; *2*) its inclusion of preschool children who were mostly zinc-deficient and stunted, a key target group; *3*) the robust sample size with a low attrition and the direct provision of the rice meals to the participating children; *4*) careful procedures to avoid zinc contamination of plasma samples; *5*) active and systematic morbidity monitoring in households; *6*) the use of sparse serial sampling to assess longitudinal changes in PZC; and *7*) the rice was consumed by the children in their household together with other usual foods, increasing generalizability. Study limitations include: *1*) the zinc-biofortification level resulted in a relatively low additional zinc intake from rice with unknown bioavailability, which might have limited the quantity of absorbable zinc and thus the effect on PZC; *2*) the household feedings of the provided rice were not supervised; thus, we cannot be sure if our estimates of rice intake are accurate because they might be overestimated (e.g., if other household members also consumed the study rice); *3*) the age range of our subjects was broad, which might have increased the variability of the response to the intervention, although subgroup analysis by age (<24 and ≥24 mo) did not change the outcome (data not shown); *4*) the secondary outcomes were not adjusted for multiplicity and thus might have resulted in increased type I error; and *5*) the PP analysis including 65% of the study population showed results comparable with the ITT analysis, which might suggest the lack of primary outcome sensitivity.

In our substudy, the putative zinc biomarker of FADS was not significantly affected. We were able to accurately measure this biomarker in a field study in a low-resource setting, despite the challenges of field collection and processing. The performance of FADS as a zinc biomarker should be further assessed in future studies of zinc fortification and supplementation.

Rice is estimated to be the major staple food crop of ∼3 billion people worldwide, providing ≤60% of their daily energy and protein intake (66). Using rice varieties with a higher genetic potential than the one used in our study, in combination with agronomic practices such as foliar spraying, could further increase zinc intake in rice-consuming populations. The practical use of such varieties and its potential impact should be further investigated.

## Supplementary Material

nqab379_Supplemental_File

## Data Availability

Data described in the manuscript, code book, and analytic code will be made available upon request pending application and approval.

## Notes

This work was supported by a grant from HarvestPlus to ETH Zurich in part by the Bill & Melinda Gates Foundation through Grant INV-002975 to HarvestPlus. HarvestPlus' principal donors are the UK Government; the Bill & Melinda Gates Foudation; the US Government's Feed the Future initiative; the Government of Canada; the European Commission; and donors to the CGIAR Research Program on Agriculture for Nutrition and Health (A4NH). HarvestPlus is also supported by the John D. and Catherine T. MacArthur Foundation.

Supplemental Methods, Supplemental Table 1, and Supplemental Figure 1 are available from the “Supplementary data” link in the online posting of the article and from the same link in the online table of contents at https://academic.oup.com/ajcn/.

MKM and RW are the senior authors.

Abbreviations used: AGP, α−1-acid glycoprotein; ARA, arachidonic acid; BFR, zinc-biofortified rice; BRRI, Bangladesh Rice Research Institute; CR, control rice; CRP, C-reactive protein; DGLA, dihomo-γ-linolenic acid; DPA, docosapentaenoic acid (22:5n–3); EED, environmental enteric dysfunction; ETH, Swiss Federal Institute of Technology; FA, fatty acid; FADS, fatty acid desaturases; GLA, γ-linolenic acid; HAZ, height-for-age *z*-score; Hb, hemoglobin; icddr, b, International Centre for Diarrhoeal Disease Research, Bangladesh; I-FABP, intestinal fatty acid binding protein; ITT, intention-to-treat; LA, linoleic acid (18:2n–6); LPR, longitudinal prevalence ratio; MEM, mixed-effects model; PA, phytic acid; PF, plasma ferritin; PP, per protocol; PZC, plasma zinc concentration; WAZ, weight-for-age *z*-score; WHZ, weight-for-height *z*-score.
