# Supplementary material for: The effect of zinc-biofortified rice on zinc status of Bangladeshi preschool children: a randomized, double-masked, household-based, controlled trial
Source: Am J Clin Nutr. 2021 Nov 18;115(3):724–37. doi: 10.1093/ajcn/nqab379 (PMC8895213; doi:10.1093/ajcn/nqab379)
Supplement: nqab379_Supplemental_File [file nqab379_supplemental_file.zip › Supplementary_Figure1_211008_v2.docx]

On-line Supplementary Material

**The effect of zinc-biofortified rice on zinc status of Bangladeshi pre-school children: a randomized, double-masked, household-based controlled trial**

Roelinda Jongstra, Md. Mokbul Hossain, Valeria Galetti, Andrew G. Hall, Roberta R. Holt, Colin I. Cercamondi, Sabina F. Rashid, Michael B. Zimmermann, Malay K. Mridha, Rita Wegmueller

**Supplementary figure 1:** Change in plasma zinc concentration (µg/dL) between baseline and endpoint in relation to baseline plasma zinc concentration (µg/dL) in Bangladeshi children, by group (control rice group n=240, biofortified rice group n=231).
