# Supplementary material for: The effect of zinc-biofortified rice on zinc status of Bangladeshi preschool children: a randomized, double-masked, household-based, controlled trial
Source: Am J Clin Nutr. 2021 Nov 18;115(3):724–37. doi: 10.1093/ajcn/nqab379 (PMC8895213; doi:10.1093/ajcn/nqab379)
Supplement: nqab379_Supplemental_File [file nqab379_supplemental_file.zip › Supplementary_methods_211008_v2.docx]

On-line Supplementary Material

**The effect of zinc-biofortified rice on zinc status of Bangladeshi pre-school children: a randomized, double-masked, household-based controlled trial**

Roelinda Jongstra, Md. Mokbul Hossain, Valeria Galetti, Andrew G. Hall, Roberta R. Holt, Colin I. Cercamondi, Sabina F. Rashid, Michael B. Zimmermann, Malay K. Mridha, Rita Wegmueller

**Supplementary methods: Detailed screening and enrolment procedure**

Before the study started, meetings were organized with local authorities and community health workers to explain the study procedures. Enrollment of children was based on a two-phase screening. In the first phase, locally recruited community health workers, conducted house visits to identify potentially eligible children between November 2017- January 2018. From the 23,372 visited households, we found 4500 children between 6-36 months of age. We used a wider age range in the first-phase screening to account for the time up to enrolment. The community health workers revisited these households to obtain the first informed consent from the caregiver, measure child’s anthropometrics (height, weight),) and record household information (caregiver’s identity, GPS location and house address) (n=3585). From the 3585 children screened, stunting prevalence (height-for-age z-score (HAZ) <-2.00) was 30% (n=1084). To include enough children in the second phase of screening, we assumed that ~40% of the children would have zinc-deficiency, and randomly selected 1014 children aged 12-36 months who had HAZ < -1.75 to the screening. The HAZ criterion was adjusted to meet our sample size calculation. This second phase screening took place after sufficient rice harvests were ensured and was based on a continuous enrollment starting April 2018. We revisited the households with the 1014 selected children, obtained a second informed consent and collected additional information on household socioeconomic status (1) and food insecurity levels (2). A health center visit was scheduled for each child in their respective unions. During the health center visit we collected a venipuncture blood sample to determined hemoglobin and plasma zinc concentration, after which eligible children were enrolled in the main study.

References

1. Browne M, Ortmann G, Hendriks SL. Household food security monitoring and evaluation using a resilience indicator: an application of categorical principal component analysis and simple sum of assets in five African countries. Agrekon 2014;53(2):25-46.

2. Swindale A, Bilinsky P. Development of a universally applicable household food insecurity measurement tool: process, current status, and outstanding issues. J Nutr 2006;136(5):1449S-52S.
