# Supplementary material for: The effect of zinc-biofortified rice on zinc status of Bangladeshi preschool children: a randomized, double-masked, household-based, controlled trial
Source: Am J Clin Nutr. 2021 Nov 18;115(3):724–37. doi: 10.1093/ajcn/nqab379 (PMC8895213; doi:10.1093/ajcn/nqab379)
Supplement: nqab379_Supplemental_File [file nqab379_supplemental_file.zip › Supplementary_Table1_211008_v2.docx]

On-line Supplementary Material

**The effect of zinc-biofortified rice on zinc status of Bangladeshi pre-school children: a randomized, double-masked, household-based controlled trial**

Roelinda Jongstra, Md. Mokbul Hossain, Valeria Galetti, Andrew G. Hall, Roberta R. Holt, Colin I. Cercamondi, Sabina F. Rashid, Michael B. Zimmermann, Malay K. Mridha, Rita Wegmueller

**Supplementary Table 1:** Comparison of plasma zinc concentration between the study groups, based on per protocol analyses. Control rice group consumed variety BRRI28, zinc-biofortified rice group consumed variety BRRI42. Total weeks of intervention n=36.

|  | Per protocol (n=337) | | | |
| --- | --- | --- | --- | --- |
|  | Regression coefficient |  | Std. Err. | p-value |
| Intercept | 4.081 | ± | 0.011 |  |
| Time | -5.26E-04 | ± | 6.30E-05 | <0.001 |
| Treatment (ref: control) | -1.87E-02 | ± | 1.50E-02 | 0.211 |
| Treatment#time (ref:control) | 1.50E-04 | ± | 8.82E-05 | 0.088 |

^1^ Mixed effect model used ln transformed plasma zinc concentrations (µg/dL) as the continuous outcome variable, time (study day from intervention start), treatment (CR versus BFR group) and their interactions as fixed effects. Subject was used as random effect. MEM contains all sampling timepoints (baseline-endpoint and two mid sparse random sampling points). Significance was set at p<0.05. BFR; zinc-biofortified rice, CR; control rice.
